# Genome-Wide Identification of the *NAC* Gene Family in *Zanthoxylum bungeanum* and Their Transcriptional Responses to Drought Stress

**DOI:** 10.3390/ijms23094769

**Published:** 2022-04-26

**Authors:** Haichao Hu, Lei Ma, Xin Chen, Xitong Fei, Beibei He, Yingli Luo, Yonghong Liu, Anzhi Wei

**Affiliations:** 1 College of Forestry, Northwest Agriculture and Forestry University, Yangling, Xianyang 712100, China; huhaichao@nwafu.edu.cn (H.H.); ml0517@nwafu.edu.cn (L.M.); cx222@nwafu.edu.cn (X.C.); feixt666@163.com (X.F.); luoyingli@nwafu.edu.cn (Y.L.); 2 Research Centre for Engineering and Technology of Zanthoxylum State Forestry Administration, Yangling, Xianyang 712100, China; 3 College of Horticulture, Northwest Agriculture and Forestry University, Yangling, Xianyang 712100, China; anning463@163.com

**Keywords:** *Zanthoxylum bungeanum*, NAC transcription factor, drought stress, gene expression, co-expression network

## Abstract

NAC (NAM, ATAF1/2, and CUC2) transcription factors (TFs) are one of the largest plant-specific TF families and play a pivotal role in adaptation to abiotic stresses. The genome-wide analysis of NAC TFs is still absent in *Zanthoxylum bungeanum*. Here, 109 ZbNAC proteins were identified from the *Z. bungeanum* genome and were classified into four groups with *Arabidopsis* NAC proteins. The 109 *ZbNAC* genes were unevenly distributed on 46 chromosomes and included 4 tandem duplication events and 17 segmental duplication events. Synteny analysis of six species pairs revealed the closely phylogenetic relationship between *Z. bungeanum* and *C*. *sinensis*. Twenty-four types of *cis*-elements were identified in the *ZbNAC* promoters and were classified into three types: abiotic stress, plant growth and development, and response to phytohormones. Co-expression network analysis of the *ZbNAC*s revealed 10 hub genes, and their expression levels were validated by real-time quantitative polymerase chain reaction (qRT-PCR). Finally, *ZbNAC007*, *ZbNAC018*, *ZbNAC047*, *ZbNAC072*, and *ZbNAC079* were considered the pivotal NAC genes for drought tolerance in *Z. bungeanum*. This study represented the first genome-wide analysis of the NAC family in *Z. bungeanum*, improving our understanding of NAC proteins and providing useful information for molecular breeding of *Z. bungeanum*.

## 1. Introduction

Transcription factors (TFs) are proteins that regulate gene expression by binding to specific *cis*-acting promoter regions of target genes [1]. The NAC (NAM, ATAF1/2, and CUC2) family is one of the largest plant-specific TF families; its name is derived from the three super families—no apical meristem (NAM), *Arabidopsis* transcription activation factor (ATAF), and cup-shaped cotyledon (CUC) [2]. NAC proteins contain a highly conserved N-terminal region (NAC domain) and a variable C-terminal region. The N-terminal domain, which consists of 150 amino acids, is further divided into 5 subdomains with diverse functions: the A subdomain is related to functional dimerization, the B and E subdomains are responsible for protein functional diversification, and the C and D subdomains are associated with DNA binding [2,3]. The variable C-terminal region of NAC TFs may also participate in transcriptional regulation through the transcriptional activation/repression region (TAR or TRR) [3].

NAC TFs have been reported to regulate various developmental processes in many plants, including lateral root construction [4], cell division [5], leaf senescence [6], fruit ripening [7], and seed germination [8]. Moreover, NAC TFs also play important roles in response to abiotic stresses such as salinity [9], low temperature [10], heat [11], and drought [12]. In recent years, drought stress has become more severe because of global warming, seriously threatening crop production and quality [13]. To date, many studies have been devoted to exploring the roles of NAC TFs in drought tolerance through molecular breeding. For example, *OsNAC2* was found to positively affect drought tolerance through ABA pathways in rice [14]. Knockdown of *TaNAC071-A* in wheat reduced plant drought tolerance, whereas its overexpression significantly enhanced drought tolerance through improved water-use efficiency [15]. Moreover, the heterologous expression of *PwNAC11* enhanced the drought tolerance in transgenic *Arabidopsis* by activating *EDR1* [16]. Furthermore, the overexpression of cowpea NAC genes, *VuNAC1* and *VuNAC2*, improved the drought tolerance of transgenic *Arabidopsis* lines [17].

*Zanthoxylum bungeanum* Maxim (2*n* = 136; genome size ~4.12 Gb), a member of the *Rutaceae* family, is an economically important plant. The *Z. bungeanum* pericarp is a popular cooking condiment and food additive in most Asian countries because of its unique taste and numbing sensation. Recent years have witnessed a rapidly increasing demand for *Z. bungeanum* pericarp due to the popularity of Sichuan hotpot, a well-known Chinese dish. In addition, *Z. bungeanum* pericarp and leaves are traditional herbal medicines for the treatment of numerous diseases; they are reported to promote blood circulation, dispel cold dampness, and diminish inflammation [18]. *Z. bungeanum* pericarp may also have significant potential in the cosmetics industry due to the high antioxidant activities of its volatile oil [19]. *Z. bungeanum* is widely cultivated in arid or semiarid areas because of its adaptation to the drought environment. Genome-wide analyses of the NAC family have been performed in a large number of species, including *Ziziphus jujuba* [20], *Helianthus annuus* [21], *Juglans mandshurica* [22], peanut [23], and sweet potato [12]. However, such an analysis has not previously been possible in *Z. bungeanum* owing to the absence of a reference genome. Notably, *Z. bungeanum* genome information was reported in our recent research [24], enabling deeper investigation into the *Z. bungeanum* plant.

In the present study, the genome-wide identification and analysis of NAC family was firstly performed in *Z. bungeanum.* Totally, 109 NAC TFs were identified from the *Z. bungeanum* genome and classified into four groups. Gene structure, conserved motif composition, chromosomal distribution, gene duplication, phylogenetic characteristics, promoter *cis*-elements, and homologous relationships among the ZbNAC TFs were investigated. In addition, the expression patterns of *ZbNAC* genes under progressive drought stress were analyzed in two *Z. bungeanum* cultivars using RNA-seq data and validated by real-time quantitative polymerase chain reaction (qRT-PCR). The results of this research are expected to improve our understanding of NAC TFs and provide valuable clues for molecular-assisted breeding in *Z. bungeanum*.

## 2. Results

### 2.1. Genome-Wide Identification, Chromosomal Distribution, Synteny Analysis, and Gene Duplication of Z. bungeanum NAC Genes

**Figure 1:**
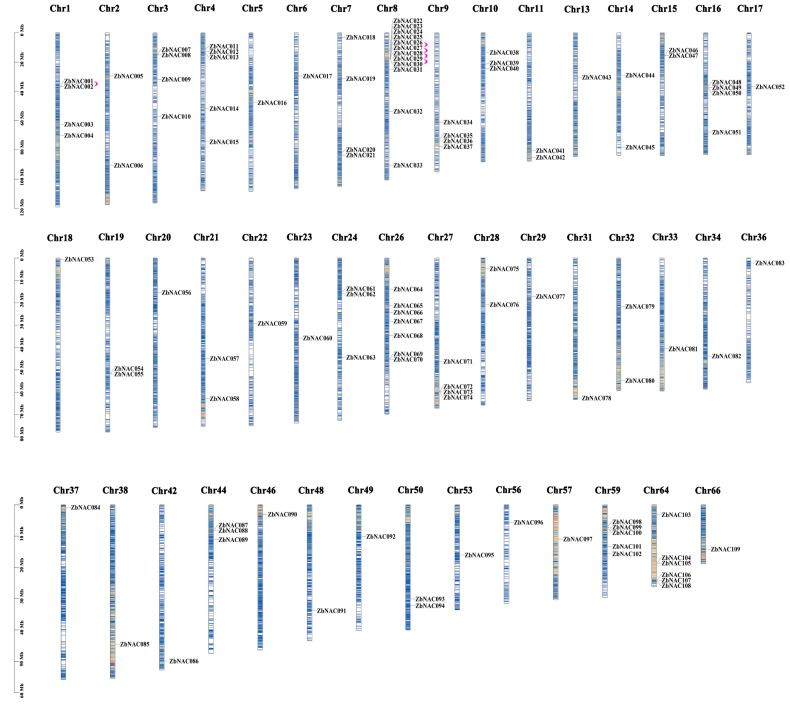
Chromosomal locations and gene duplications of the *ZbNAC* genes. All 109 *ZbNAC* genes are shown on the chromosomes and indicated by their names. Chromosome numbers are presented at the top of each bar. Tandemly duplicated gene pairs are indicated with pink brackets.

In total, 109 non-redundant genes containing NAC domains were identified in *Z. bungeanum* (*ZbNAC*s). All were located alone or in clusters on 46 chromosomes, and they were named based on their genetic locations across the *Z. bungeanum* chromosomes (*ZbNAC001*–*ZbNAC109*) (Figure 1). The number of ZbNAC genes varied among chromosomes, with the largest number of NAC genes (12) on chromosome 8 and a single NAC gene on 23 chromosomes. The physical and chemical properties of the *ZbNAC* genes are listed in Table S1. The coding sequences (CDSs) ranged from 579 to 2307 bp, and the predicted proteins varied from 192 to 768 amino acids in length, with molecular weights between 48.18 and 193.36 kDa. Their theoretical isoelectric points (pIs) varied from 4.90 to 5.19, with an average of 5.07, indicating that most ZbNAC proteins were weakly acidic. Subcellular location prediction suggested that most ZbNAC proteins were located in the nucleus (81), some in the plasma membrane (31) and chloroplast (15), and a few in the cytoplasm (5), peroxisome (4), and Golgi apparatus (1). These subcellular location results suggest that ZbNAC TFs may participate in the regulation of nuclear genes and have multiple functions in adaptation to diverse environments.

**Figure 2:**
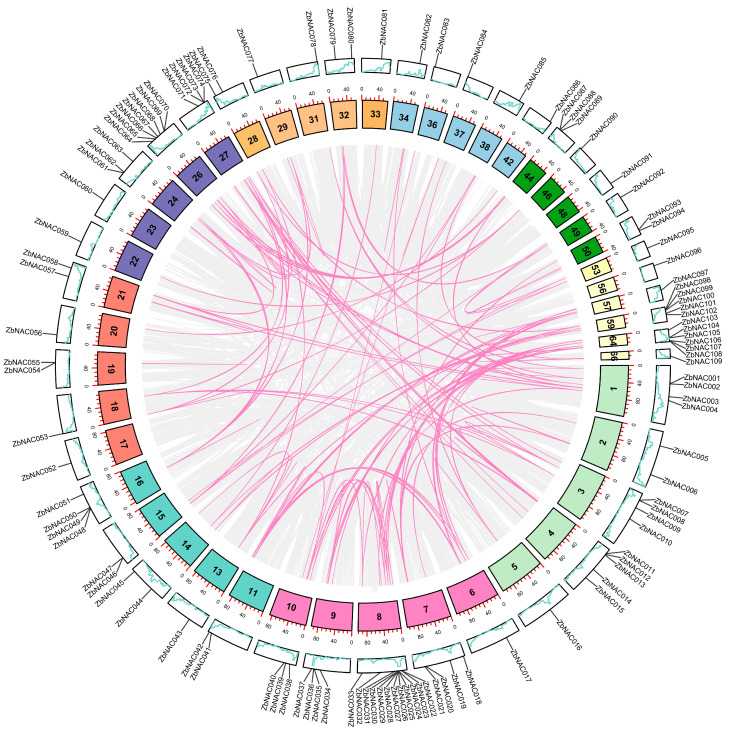
Collinearity analysis of 109 *ZbNAC* genes. The lines inside the circle indicate collinear blocks within the *Z. bungeanum* chromosomes. The pink lines indicate segmental duplication events related to *ZbNAC* genes. The numbers in the inner blocks are the chromosome numbers. The green lines in the outer blocks indicate gene density, with higher values indicating higher density.

Duplication events were analyzed in the 109 *ZbNAC* genes (Table 1, Figure 2). Five pairs of tandem duplicates were distributed on two chromosomes, Chr 1 and Chr 8. Interestingly, four pairs of tandem duplications were located on Chr 8 and consisted of five adjacent *ZbNAC* genes, indicating that these five *ZbNAC* genes probably share a common ancestor. In addition, 42 pairs of segmental duplications were identified among the 109 *ZbNAC* genes. These results suggest that segmental duplication may have been the main driving force during ZbNAC family evolution. Ka and Ks can be used to measure the course of divergence, and the *Ka*/*Ks* ratio can be used as a criterion for positive selection pressure after duplication. Specifically, *Ka*/*Ks* = 1, <1, and >1 represent neutral selection, purifying selection, and accelerated evolution under positive selection, respectively. The *Ka*/*Ks* values for segmental duplicates ranged from 0.11 to 1.13 with an average of 0.40, and those for tandem duplicates ranged from 0.24 to 0.81 with an average of 0.72. Only one tandem duplicate and one segmental duplicate showed *Ka*/*Ks* > 1. These results suggest that most *ZbNAC*s evolved primarily under purifying selection.

**Figure 3:**
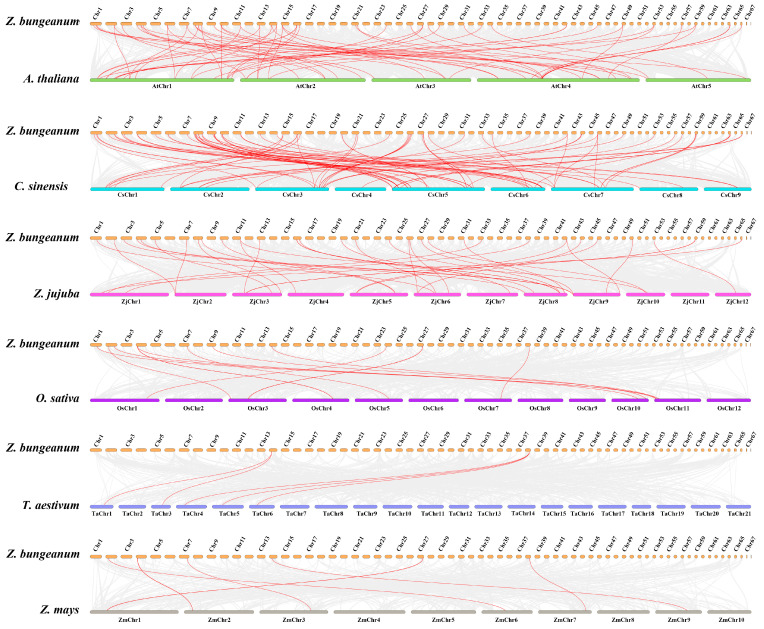
Synteny analysis of *NAC* genes between *Z. bungeanum* and *A. thaliana*, *C. sinensis*, *Z. jujube*, *O. sativa*, *T. aestivum*, and *Z. mays.* Gray lines in the background represent collinear relationships between *Z. bungeanum* and six species, and the red lines represent the collinear *NAC* gene pairs.

To further explore the evolutionary relationships between *Z. bungeanum* and other plants, we constructed six collinearity maps of NAC genes for *Z. bungeanum*, *A. thaliana*, *Camellia sinensis*, *Ziziphus jujube*, *Oryza sativa*, *Triticum aestivum*, and *Zea mays* (Figure 3). There were 41, 72, and 39 orthologous gene pairs between *Z. bungeanum* and *A. thaliana*, *C. sinensis*, and *Z. jujube*. However, there were fewer orthologous pairs between *Z. bungeanum* and *O. sativa* (14), *T. aestivum* (5), and *Z. mays* (8).

### 2.2. Phylogenetic Analysis of ZbNAC Proteins

**Figure 4:**
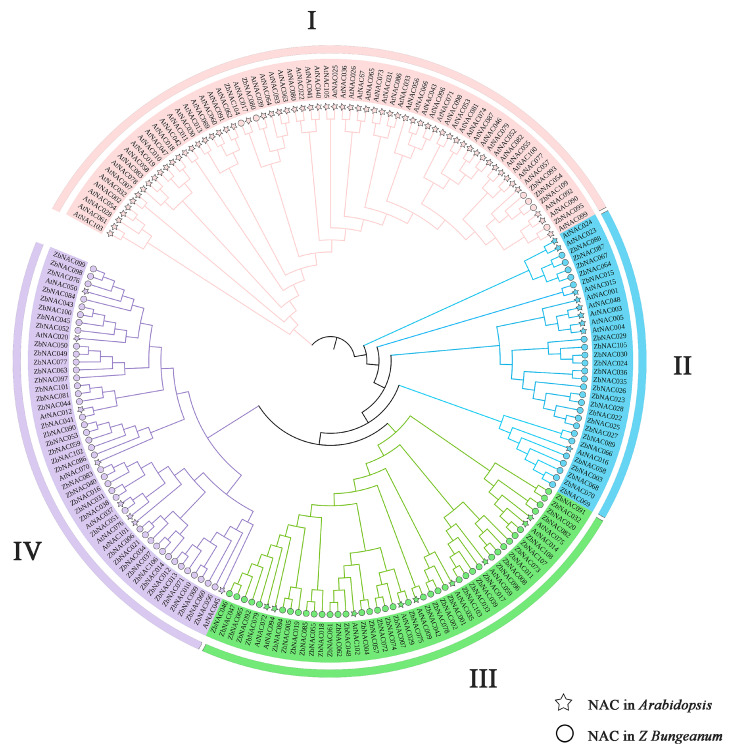
Phylogenetic relationships of NAC transcription factors (TFs) from *Z. bungeanum* and *A. thaliana*. The phylogenetic tree was constructed using the neighbor-joining method with 1000 bootstraps in MEGA 7.0 software. Various background colors show the NAC protein groups. The NAC TFs in *A. thaliana* are indicated by stars, and the NAC TFs in *Z. bungeanum* are indicated by circles.

To explore the evolutionary relationships among NAC proteins, a phylogenetic reconstruction was performed based on a multiple sequence alignment using MEGA 7.0 (Figure 4). An unrooted phylogenetic tree was constructed with 109 ZbNAC TFs and 88 AtNAC TFs based on the conserved NAC domain, and the proteins were clustered into four distinct groups (I–IV). Group I contains the most AtNAC members (62) and the fewest ZbNAC members (6), suggesting that there may be a divergence from the last common ancestor in this group. There were fewer NAC members in *A. thaliana* than in *Z. bungeanum* in groups II–IV: group II contains 9 AtNAC proteins and 24 ZbNAC proteins, group III contains 9 AtNAC proteins and 38 ZbNAC proteins, and group IV contains 8 AtNAC proteins and 41 ZbNAC proteins. ZbNAC and AtNAC proteins with close phylogenetic relationships may have similar gene structures and biological functions.

### 2.3. Gene Structures and Conserved Motifs in ZbNAC Proteins

**Figure 5:**
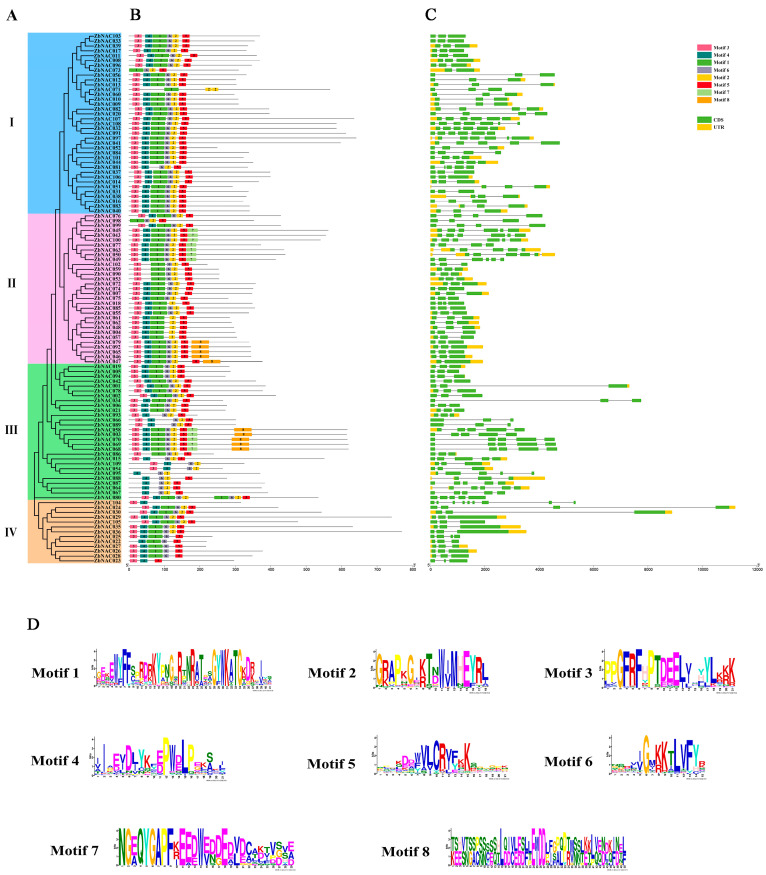
Phylogenetic tree, conserved motifs, and genetic structures of 109 ZbNAC TFs. (**A**) Phylogenetic tree of 109 ZbNAC TFs. (**B**) Conserved motifs in the 109 ZbNAC proteins. Different motifs are presented in different colored boxes. (**C**) Gene structures of *ZbNAC* genes. Exons and untranslated regions (UTRs) are indicated by green and yellow boxes, and black lines indicate introns. The ruler at the bottom is used to indicate length. (**D**) The sequence logos of eight motifs. The height of each letter indicates the degree of conservation. Numbers on the *x*-axis represent residue positions, and the *y*-axis represents the content measured in bits.

To further explore the relationships among ZbNAC family members, the phylogeny, gene structures, and conserved motifs of the ZbNAC TFs were analyzed (Figure 5). Eight conserved motifs were identified in the 109 ZbNAC proteins, and their amino acid lengths varied from 15 to 50 (Figure 5B,D). The motif numbers in the ZbNAC proteins varied from 2 (ZbNAC104) to 9 (ZbNAC080). Motifs 1–6 were found in the NAC domain regions of 79 ZbNAC proteins. Motif 5 may be the most important motif in the ZbNAC TFs, as it was present in almost all proteins except for ZbNAC109, ZbNAC054, ZbNAC071, and ZbNAC095. Conversely, Motif 8 was least common and was present in only 10 ZbNAC proteins. Although motif types and numbers differed among ZbNACs from different groups, most members with a close phylogenetic distance shared the same motifs and similar motif locations.

Gene structure analysis showed that the ZbNAC encoding genes contained 1 to 8 introns (Figure 5C). Most of the *ZbNAC* genes (67) contained two introns, two (*ZbNAC073*, *ZbNAC047*) contained one intron, and one (*ZbNAC080*) contained eight introns. As expected, genes that were closely phylogenetically related shared common motifs and similar motif locations. For example, *ZbNAC068*, *ZbNAC069*, and *ZbNAC070* are clustered together in the phylogeny tree; all have five exons with similar locations and lengths.

### 2.4. Cis-element Analysis of ZbNAC Genes

**Figure 6:**
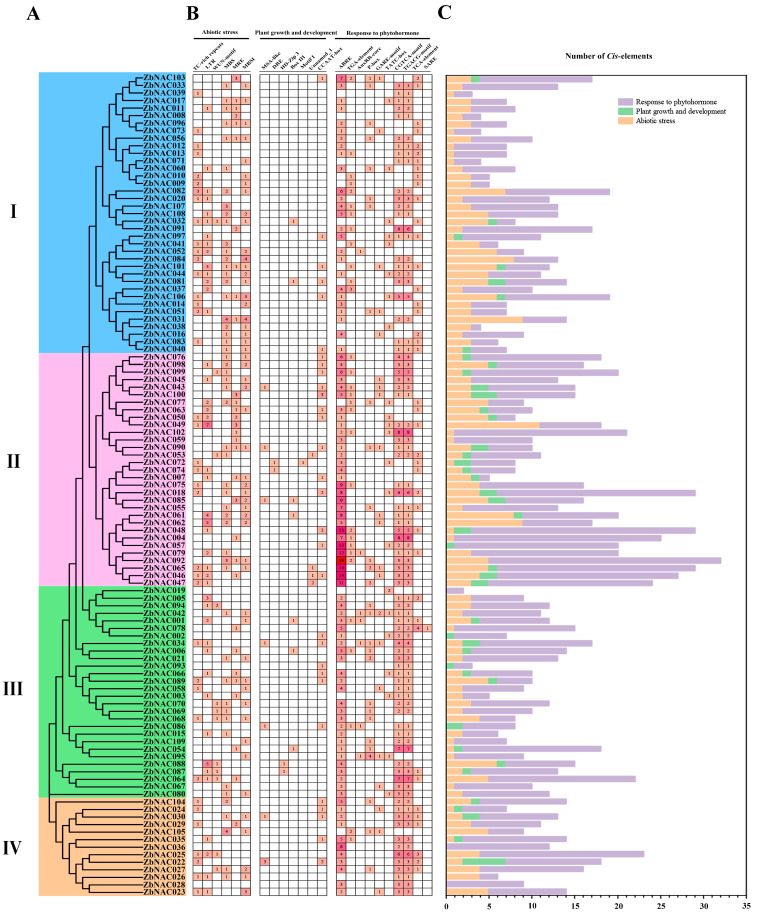
Analysis of specific *cis*-elements in *ZbNAC* promoters. (**A**) Phylogenetic tree of 109 *ZbNACs*. (**B**) Statistics for *cis*-acting elements in the promoter regions of *ZbNAC* genes. The numbers of specific *cis*-elements are indicated by numbers and a red color gradient. (**C**) Color-coded histograms indicate the numbers of *cis*-elements in each category.

Twenty-three types of *cis*-elements were distributed unevenly in the 2000-bp upstream regions of the 109 *ZbNAC* genes (Figure 6 and Figure S1). These *cis*-elements were classified into three categories: abiotic stress (6 types), plant growth and development (7 types), and response to phytohormones (10 types). Judging from the numbers of these elements, they are mainly involved in phytohormone pathways and responses to abiotic stress, thus revealing the important role of *ZbNAC*s in the regulation of hormone synthesis and adaptations to environmental stress. With respect to abiotic stress, TC-rich repeats (75) were the most abundant elements; they were located in 45 *ZbNAC* promoters and were annotated as drought response elements (Figure S2). With respect to hormone response, the *cis*-elements were mainly involved in abscisic acid, auxin, gibberellin, methyl jasmonate (MeJA), and salicylic acid pathways. Among these elements, ABRE elements were the most abundant (369, abscisic acid responsive) followed by CGTCA motifs (206, MeJA responsive) and TGACG motifs (206, MeJA responsive). The numbers and types of *cis*-elements were similar in *ZbNAC* genes that had a close phylogenetic relationship. In group I, there were fewer elements, and they were seldom assigned to the plant growth and development category. In group II, the number of *cis*-elements was highest, and TC-rich repeats, ABRE elements, CGTCA motifs, and TGACG motifs were the most abundant. For example, there were 18 ABRE elements in *ZbNAC092*, 14 ABRE elements in *ZbNAC065* and *ZbNAC046*, and 8 CGTCA motifs in *ZbNAC004* and *ZbNAC102*, and these elements were annotated as ABA and MeJA responsive. The ZbNAC TFs in group II may therefore be important for drought response and ABA and MeJA regulation.

### 2.5. RNA-seq Analysis of 109 ZbNAC Genes under Drought Stress

**Figure 7:**
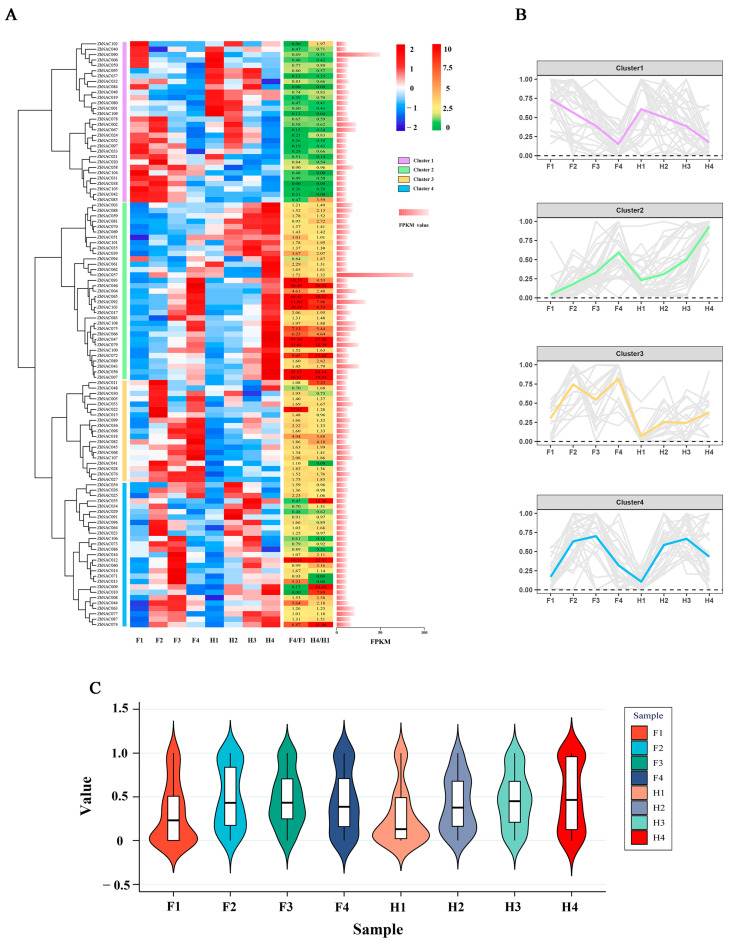
The expression patterns of 109 *ZbNAC* genes evaluated by RNA-seq. (**A**) Hierarchical clustering heat maps based on FPKM values of 109 *ZbNACs*. Four clusters are indicated by different colors on the left. The left heatmap shows gene expression as FPKM, with high levels in red and low levels in blue. The right heatmap shows the FPKM ratios of F4/F1 and H4/H1, with high ratios in red and low ratios in green. The right horizontal bar chart shows the mean FPKM value of each gene across all samples. (**B**) Gene expression trends of the four clusters. (**C**) Violin diagrams of FPKM values in different samples. F1, F2, F3, and F4 indicate FJ samples on days 0, 6, 9, and 15, respectively; H1, H2, H3, and H4 indicate HJ samples on days 0, 6, 9, and 15, respectively.

A hierarchical clustering heatmap was used to visualize the expression patterns of 109 *ZbNAC* genes of two *Z. bungeanum* cultivars under drought stress. These genes were divided into four clusters (clusters 1–4) based on their FPKM values (Figure 7A,B). In cluster 1, the total expression level of 30 *ZbNAC*s decreased with progressive drought stress in both FJ and HJ, and the F4/F1 and H4/H1 ratios were less than one, with the exception of *ZbNAC085* (3.59) and *ZbNAC102* (1.97) in HJ. Thirty-three *ZbNAC*s were placed into cluster 2, and most of them increased in expression with progressive drought stress, with higher expression in HJ than in FJ. The F4/F1 and H4/H1 ratios were greater than one in almost all members of cluster 2 except for *ZbNAC081* (0.95) and *ZbNAC094* (0.84). Interestingly, the ratios of six *ZbNAC*s from cluster 2 (*ZbNAC007, ZbNAC046*, *ZbNAC047*, *ZbNAC056*, *ZbNAC065*, and *ZbNAC079*) were greater than ten in both cultivars, suggesting that these six genes may play pivotal roles in response to drought stress. There were 19 *ZbNAC*s in cluster 3; most were significantly enhanced in FJ during early drought, but the changes in HJ were less marked, indicating that the genes in cluster 3 were specifically involved in adaptation to drought stress in FJ. In cluster 4, the expression levels of most genes increased in the early drought period and decreased in the later period, suggesting that these TFs may regulate the expression of early drought-responsive genes in *Z. bungeanum*. In addition, the average FPKM values suggested that the expression levels of *ZbNAC057* (99.52) and *ZbNAC090* (44.15) were significantly higher than those of other genes, which averaged 4.30. The total expression levels of the 109 *ZbNAC* genes were displayed in violin diagrams (Figure 7C). Most genes had a low expression level under control conditions and were significantly induced under drought stress. Taken together, the 87 *ZbNAC* genes in clusters 2, 3, and 4 may positively regulate the drought tolerance of *Z. bungeanum* and were therefore selected for further study.

### 2.6. Comprehensive Analysis of Drought-Related ZbNAC Genes

**Figure 8:**
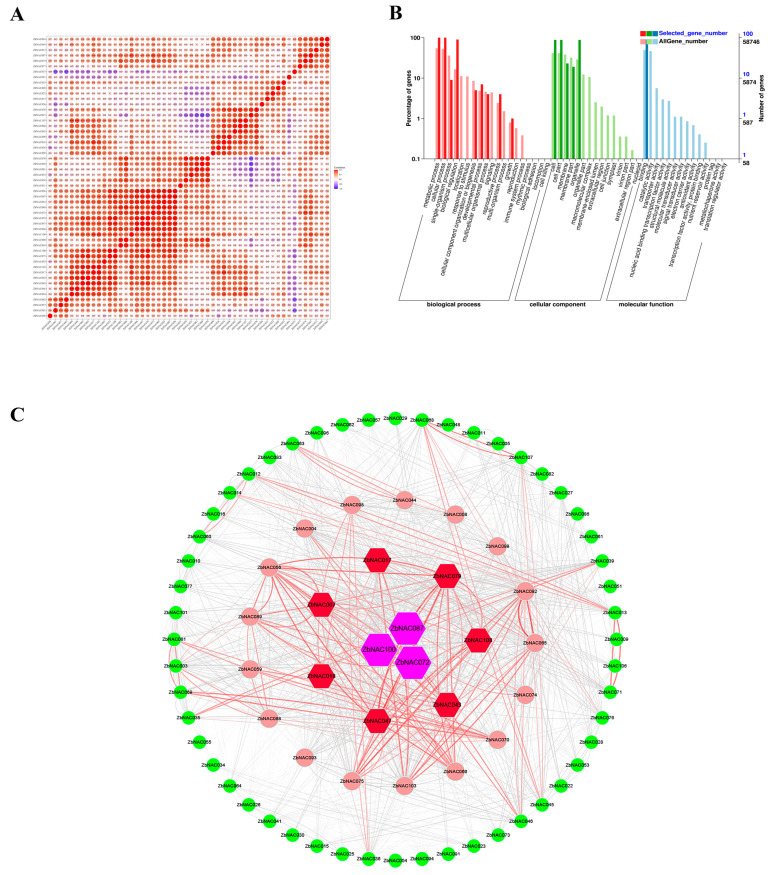
Comprehensive analysis of drought-related *ZbNAC* genes. (**A**) Correlation analysis of upregulated *ZbNAC*s. Red indicates a positive correlation, and blue indicates a negative correlation. The circle size indicates the absolute value of the correlation coefficient. × indicates that the correlation is not significant. (**B**) GO enrichment analysis of upregulated *ZbNAC*s. (**C**) Co-expression network analysis. The red line indicates a significant positive correlation (*p* < 0.01). The size indicates the degree calculated by the Cytohubba program. The ten polygons situated in the center represent hub genes with high connectivity.

Correlation analysis was performed for 87 genes in clusters 1, 2, and 3 with FPKM values greater than 1 to explore their possible relationships (Figure 8A). The results showed that 742 pairs of genes showed positive correlations (*p* < 0.05), and only 12 pairs showed negative correlations (*p* < 0.05). GO analysis assigned GO terms to these genes from three categories: biological process (BP), cellular component (CC), and molecular function (MF) (Figure 8B). Among BP terms, metabolic process, cellular process, and biological regulation were most common; among CC terms, cell, cell part, and organelle were most common; only one MF term, binding, was assigned to the selected genes. A co-expression network was then constructed based on Pearson correlation coefficients. Ten hub genes (*ZbNAC007*, *ZbNAC017*, *ZbNAC018*, *ZbNAC043*, *ZbNAC047*, *ZbNAC072*, *ZbNAC079*, *ZbNAC087*, *ZbNAC100*, and *ZbNAC108*) were identified using the Cytohubba program in Cytoscape 3.9.0 (Figure 8C). Three hub genes (*ZbNAC087*, *ZbNAC072*, and *ZbNAC100*) were the most dominant in the network because of their high connectivity with other genes.

**Figure 9:**
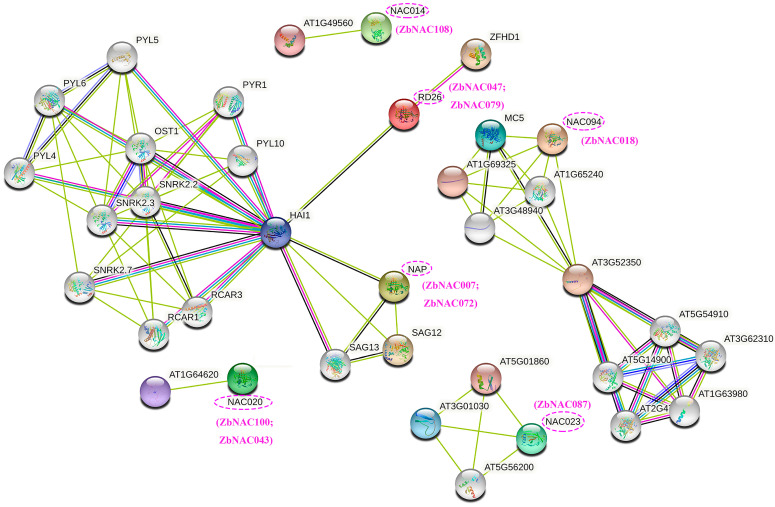
Protein–protein interaction network of *Arabidopsis* NAC homologs predicted with STRING 11.5.

**Figure 10:**
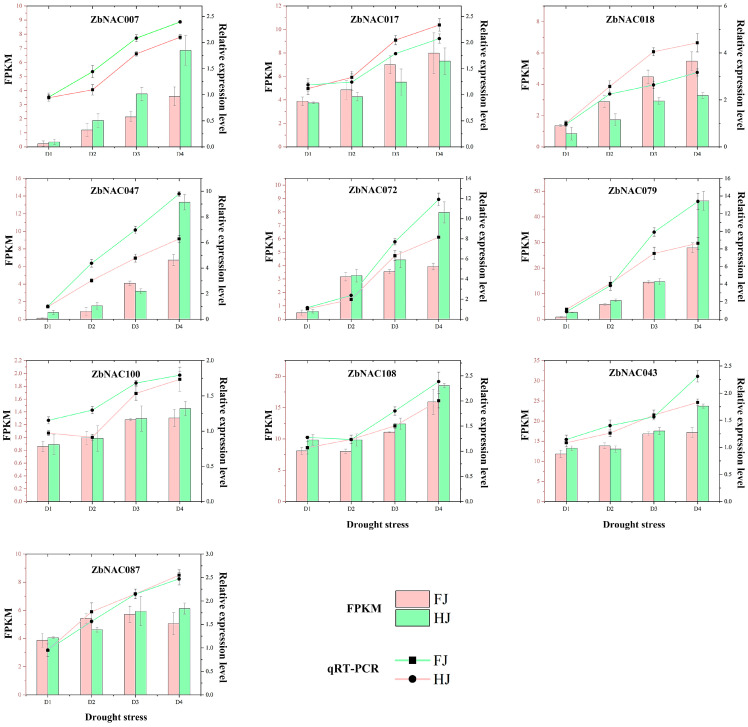
Comparison of expression levels measured by RNA-seq and qRT-PCR for ten *ZbNAC* hub genes under drought stress. The RNA-seq expression levels are shown in bar charts based on FPKM values; the relative expression levels measured by qRT-PCR are displayed as line graphs.

The AtNAC homologs of the ten hub ZbNAC proteins were predicted from the phylogenetic tree (Figure 1) and are listed in Table S2. To further examine the functions of the ten hub genes, a protein–protein interaction network of ten hub ZbNAC proteins was predicted based on their AtNAC homologs using STRING (Figure 9). NAC094 (homolog of ZbNAC018) was directly connected to MC5, with a score of 0.909. RD26 (homolog of ZbNAC047 and ZbNAC079) was directly connected to ZFHD1 (0.860) and HAI1 (0.804). NAP (homolog of ZbNAC007 and ZbNAC072) was directly connected to SAG12 (senescence-associated gene 12 encoding protein), SAG13 (senescence-associated gene 13 encoding protein), and HAI1 (highly ABA-induced 1). Notably, HAI1 was involved in a cluster network that also contained genes encoding sucrose non-fermenting–related kinase 2 (SnRK2), PYR, ABA receptors (PYLs), regulatory components of ABA receptor (RCAR), and open stomata 1 (OST). GO analysis annotated the genes in the predicted network with 32 GO terms, including abscisic acid binding, signaling receptor activity, abscisic acid-activated signaling pathway, response to abscisic acid, and signal transduction (Table S3). KEGG analysis showed that 14 genes were annotated with the pathways mitogen-activated protein kinase (MAPK) signaling pathway-plant and plant hormone signal transduction (Table S4). Moreover, qRT-PCR showed that the relative expression levels of the ten hub genes increased under drought stress (Figure 10), and the expression data from qRT-PCR were consistent with the RNA-seq data.

## 3. Discussion

NAC TFs are one of the largest plant-specific TF families and play significant roles in plant growth, development, and adaptation to environmental stress [25]. Genome-wide identification of NAC family members has been performed in many plant species but has not yet been available in *Z. bungeanum* owing to the absence of a reference genome. Recently, a chromosome-scale assembly of *Z. bungeanum* genome with high quality was published in our research [24], which provided the valuable opportunities for the further study of *Z. bungeanum*. Here, we firstly performed the comprehensive genome-wide identification of NAC family members and characterized their gene expression under drought stress in *Z. bungeanum*.

Based on the presence of the NAC domain structure, 109 non-redundant ZbNAC TFs were identified, close to the 108 NACs in *Dactylis glomerata* [26]; more than the 82 in *Cucumis melo* [27], 80 in *Fagopyrum tataricum* [28], and 96 in *Manihot esculenta* [29]; and fewer than the 151 in *O. sativa* [30], 152 in *Z. mays* [31], and 152 in *Glycine max* [32]. These results suggest that the evolutionary expansion of the NAC family differed among species. There are a large number of chromosomes (*n* = 68) in the *Z. bungeanum* genome, owing to frequent fusion/fission events after whole-genome duplication [24]. The 109 NAC genes are distributed unevenly on most of the chromosomes (46/68), and this may be closely related to their functional diversity.

Gene duplication is a potential driving force for species evolution as well as gene functional diversity [33]. NAC TFs in 160 plant species are reported to have evolved from common ancestors and to have undergone numerous duplication events [34]. Segmental and tandem duplications are considered the main types of duplication events in eukaryotes [35]. In general, more segmental duplications than tandem duplications have been found in the NAC family, such as the 84 segmentally duplicated pairs and 5 tandemly duplicated pairs in *Panicum miliaceum* [36], the 17 segmentally duplicated pairs and 2 tandemly duplicated pairs in *Musa acuminata* [37], and the 116 segmentally duplicated pairs and 1 tandemly duplicated pair in peanut [23]. In *Z. bungeanum*, 42 pairs of segmental duplicates and 5 pairs of tandem duplicates were identified, indicating that segmental duplication was more common during evolution of the *ZbNAC*s. Interestingly, five tandem duplicates were found together on chromosome 2. We speculate that these five genes may have a common ancestor and may therefore share similarities in structure and function. *Ka*/*Ks* = 1, <1, and >1 are indicative of neutral selection, purifying selection, and accelerated evolution under positive selection, respectively [38]. In this research, most duplicates showed *Ka*/*Ks* <1, indicating that most *ZbNAC*s evolved primarily under purifying selection. *C. sinensis*, *Z. bungeanum*, *Z. jujube*, and *Arabidopsis* are dicots, whereas maize, rice, and wheat are monocots. Here, the dicots exhibited greater collinearity with *Z. bungeanum* than the monocots, indicating that NAC TFs experienced extensive evolution and duplication after the divergence of monocots and dicots. Feng et al. [24] found that *Z. bungeanum* shared a close phylogenetic relationship with *C. sinensis* and that they diverged from their most recent common ancestor approximately 35.3 million years ago. Consistent with this finding, the greatest number of collinear NAC genes were found between these two species.

Exons and introns are important features of gene evolution, and exons are an important part of protein-coding genes [39]. The insertion or deletion of introns can result in a loss of function or gene mutation. In addition, genes with fewer introns may respond more quickly, and genes with large-scale introns possibly have specific functions [32]. The number of introns in the *ZbNAC* genes varied from 1 to 8, similar to numbers reported in chickpea (1–6) [40] and sunflower (2–7) [41]. Family members with close phylogenetic relationships generally have similar gene structures [42], and this phenomenon was also found in our research. For instance, *ZbNAC068*, *ZbNAC069*, and *ZbNAC070*, which were most closely related in the same group, harbored five exons of similar location and length. The two *ZbNAC* genes with only one intron (*ZbNAC073* and *ZbNAC047*) may have a faster response speed and may be located upstream in the response pathway. *ZbNAC080* contained eight introns, and the length of each exon was relatively short. Thus, we suspect that the insertion of introns led to mutation in gene structure and that *ZbNAC080* may have specific functions. The motifs in TF proteins are often associated with DNA binding, protein interaction, and transcriptional activity [28,43]. A total of 10 conserved motifs were identified in the 109 ZbNAC TFs. Motifs 1–5 were present in almost all NAC members in groups 1–3, indicating that they probably have significant biological functions. Motif 5 was the most common, suggesting that it may be located in a conserved domain of the ZbNAC proteins. All the conserved motifs were located in the N termini of the protein sequences, indicating that their existence is essential to NAC function [2].

The structure and regulatory mechanisms of gene promoters are closely related to specific plant traits [44]. The physiological functions of promoter *cis*-elements indicate possible responses of these genes to internal and external factors [44]. Twenty-four types of promoter elements were identified in *ZbNAC*s and were divided into three categories: abiotic stress response elements, hormone response elements, and developmental regulation elements. Numerous elements were involved in abiotic stress and hormone response, consistent with NAC genes in jujube [20], implying that most *ZbNAC*s may be associated with plant hormone signaling and abiotic stress responses. LTR (response to low temperature) and CCTG (response to drought stress) accounted for a large proportion of abiotic stress elements, suggesting a vital role for *ZbNAC*s in low temperature and drought stress. Consistent with this notion, substantial evidence suggests that the NAC gene family plays a critical role in plant responses to abiotic stress. For example, overexpression of *ONAC022* improved drought and salt tolerance in rice [45], and overexpression of *MbNAC25* from *Malus baccata* increased the resistance of transgenic *Arabidopsis* to cold and salinity [46]. There were large numbers of ABA elements and MeJA elements in the *ZbNAC* promoters. Likewise, ABA and MeJA elements were also abundant in NAC promoters of *Ipomoea trifida* [12]. ABA and MeJA are well known as important phytohormones for plant response and adaptation to various abiotic stresses, including salinity, drought, and cold [47]. Previous research has shown that ABA and MeJA are easily induced by drought stress [48], and exogenous application of ABA and MeJA can enhance plant drought tolerance [49,50,51]. The results of this study suggest that most *ZbNAC*s may participate in drought resistance via regulation of ABA and MeJA.

*Z. bungeanum* is a commercial crop that is widely grown in arid and semi-arid regions, and the above analyses revealed the possible function of *ZbNAC*s in drought response. Therefore, exploring the expression of these *ZbNAC*s under drought is necessary for a comprehensive analysis of the ZbNAC family. In the present research, RNA-seq was performed with two *Z. bungeanum* cultivars of different drought tolerance under progressive drought stress. The 109 *ZbNAC* genes were divided into four groups based on their expression levels, and three clusters (79 genes, 70%) were upregulated by drought, suggesting that most *ZbNAC* genes are involved in drought tolerance of *Z. bungeanum*. Likewise, drought also increased the expression levels of most NAC genes in peanut [52] and pepper [53]. Correlation analysis showed that the majority of upregulated genes were significantly positively correlated, suggesting that they may play a synergistic role in drought tolerance. GO analysis showed that their encoded proteins were involved in binding, indicating that NAC genes may regulate the drought response by binding to target genes. Co-expression analysis has been widely used to screen key genes from large gene sets [54,55,56]. Here, ten hub genes with a high degree of connectivity to other genes were identified in the constructed co-expression network. We speculate that these ten *ZbNAC*s are pivotal genes that dominate the interactions of transcription factors, and they may have important regulatory functions in drought response. The qRT-PCR results showed that the expression levels of these ten *ZbNAC*s increased with drought, and most of their expression levels were higher in HJ than in FJ. This result not only verified our speculation but also confirmed the reliability of our RNA-seq data.

Plant drought response mechanisms are complex and include ROS scavenging, stomatal closure, root elongation, production of antioxidant enzymes, and accumulation of antioxidant substances [57]. MAPK and ABA signaling pathways play an important role in plant responses to abiotic stress, including drought stress and salt stress [58]. Under drought stress, plants perceive the drought signal by the ABA signaling pathway, which consists of PYLs, Clade A protein phosphatase 2Cs (PP2Cs), and SnRK2 [59]. PYL–PP2C signaling can activate MAPK signaling cascades to respond to drought stress [60]. Our protein–protein interaction network predicted that NAP (homolog of ZbNAC007 and ZbNAC072) and RD26 (homolog of ZbNAC047 and ZbNAC079) interacted with HAI1, a member of the PP2C family. HAI1 could interact with SNRK2.2, SNRK 2.3, PYL4, RCAR3, and OST1. In addition, KEGG enrichment analysis showed that the target genes of these NAC TFs were enriched in the MAPK and ABA signaling transduction pathways. These results indicate that NAP and RD26 may regulate drought tolerance by affecting the MAPK and ABA signal transduction pathways. Consistent with this notion, previous reports indicate that NAP and RD26 mediate drought stress–responsive signaling in *Arabidopsis* [61,62]. The protein–protein interaction network also showed that NAC094 (ZbNAC018 homolog) could interact with MC5, which is associated with programmed cell death (PCD) under abiotic stress [63]. PCD has been reported to promote the development of lateral roots with enhanced drought tolerance, which are important for post-stress recovery [64]. Thus, the NAC094 protein may also be important for drought tolerance. Because of the conservation of NACs, NAC proteins encoded by homologous genes usually share common biological functions [11,21]. Consequently, the corresponding homologous genes *ZbNAC007*, *ZbNAC047*, and *ZbNAC072*, and *ZbNAC079* may participate in the regulation of MAPK and ABA signaling transduction pathways, and *ZbNAC018* may be involved in PCD in *Z. bungeanum* under drought. With regard to subcellular localization, these five ZbNAC proteins were all located in the nucleus, further emphasizing their importance in regulating the expression of target genes under drought stress. Taken together, *ZbNAC007*, *ZbNAC018, ZbNAC047*, *ZbNAC072*, and *ZbNAC079* are potential candidate genes for improving the drought resistance of *Z. bungeanum*, but further verification research needs to be performed in the future.

## 4. Materials and Methods

### 4.1. Identification of NAC Genes in Z. bungeanum

The *Z. bungeanum* genome resource was published in our previous research (BioProject ID PRJNA524242) [24]. The hidden Markov model (HMM) file of the NAM domain (PF02365) was obtained from the Pfam database (http://pfam.sanger.ac.uk/, accessed on 12 February 2022). HMMER 3.0 was used to identify the NAC proteins in the *Z. bungeanum* genome with a cut-off value of 0.01 [65]. Redundant NAC proteins were deleted using an online tool (https://web.expasy.org/decrease_redundancy, accessed on 12 February 2022). Subsequently, the putative NAC proteins were further confirmed by BLASTP and the Conserved Domain Database (CDD, http://www.ncbi.nlm.nih.gov/cdd/, accessed on 12 February 2022). Protein physicochemical parameters, including number of amino acids, molecular weight, and pI value, were predicted using ExPasy website tools (http://web.expasy.org/protparam/, accessed on 12 February 2022). The subcellular localization of the NAC proteins was predicted using WoLF PSORT (https://wolfpsort.hgc.jp/, accessed on 12 February 2022) [66].

### 4.2. Chromosomal Distribution and Gene Duplication

The chromosomal positions of the *ZbNAC* genes were extracted from the GFF file of the *Z. bungeanum* genome. The chromosomal map with gene positions was produced using TBtools (v. 1.098696) [67]. The *ZbNAC* genes were named according to their chromosomal positions, *ZbNAC001*–*ZbNAC109*. MCScanX software with default parameters was used to analyze gene duplication events among 109 *ZbNAC* genes [68]. The genome file for rice was downloaded from Phytozome (http://phytozome.jgi.doe.gov/pz/portal.html#, accessed on 15 February 2022) [69], and the *Arabidopsis* genome file was obtained from the *Arabidopsis* Information Resource (https://www.arabidopsis.org/, accessed on 15 February 2022). The genome sequences and annotation files of wheat, maize, and jujube were acquired from NCBI (https://www.ncbi.nlm.nih.gov/, accessed on 16 February 2022). The synteny analysis between *Z. bungeanum* and other species was performed using TBtools (v. 1.098696, JAVA, China), and with parameters CPU for BLATP: 4, evalue: 1× 10^-10^, Num of BlastHits: 5.

### 4.3. Sequence Alignment and Phylogenetic Analysis of ZbNAC TFs

The NAC protein sequences from *Z. bungeanum* and *Arabidopsis* were aligned using ClustalW in MEGA 7.0, and an unrooted phylogenetic tree was constructed using the neighbor-joining (NJ) method in MEGA 7.0 with pairwise deletion and 1000 bootstrap replicates. The resulting phylogenetic tree was further processed with the online tool iTOL (https://itol.embl.de/, accessed on 18 February 2022).

### 4.4. Gene Structure, Conserved Motif, and Cis-element Analyses of the ZbNAC TFs

The online Gene Structure Display Server 2.0 (GSDS, http://gsds.gao-lab.org/, accessed on 18 February, 2022) was used to determine exon–intron structures by comparing CDSs and genomic sequences of the *ZbNAC*s. Conserved motifs were identified in the ZbNAC proteins using the online program MEME (http://meme-suite.org/tools/meme, accessed on 18 February 2022) with a maximum of 10 motifs and a range of motif widths from 6 to 200. The integrative analysis of gene structure and conserved motifs was visualized using TBtools [67].

The 2000-bp sequences upstream of the *ZbNAC* start codons were extracted and submitted to PlantCARE (http://bioinformatics.psb.ugent.be/webtools/plantcare/html/, accessed on 22 February 2022) for *cis*-element analysis. The PlantCARE output file was used to illustrate the *cis*-element distribution in the *ZbNAC* promoters using TBtools.

### 4.5. Plant Materials and RNA-seq

Mature seeds of FJ (*Z. bungeanum* cv. ‘Fengjiao’) and HJ (*Z. bungeanum* cv. ‘Hanjiao’) were collected from the *Z. bungeanum* Experimental Station of Northwest Agriculture and Forestry University in Fengxian, Shaanxi province, China (33°59′6.55′′ N, 106°39′29.38′′ E). The seeds were sown in a mixture of perlite, vermiculite, and chernozem, and the resulting seedlings were cultivated at 25 ± 2 °C and 60–70% humidity in an experimental greenhouse at Northwest Agriculture and Forestry University in Yangling, Shaanxi province, China. Three months after germination, healthy seedlings of similar size were selected for drought treatment. After stopping the water supply, leaves were sampled at 0 d (D1), 6 d (D2), 9 d (D3), and 15 d (D4), frozen in liquid nitrogen, and stored in a −80 °C freezer.

Biomarker Technologies Co., Ltd. (Beijing, China) performed the RNA-seq analysis of 24 *Z. bungeanum* samples. The cDNA libraries were built following the manufacturer’s instructions of the NEBNext Ultra RNA Library Prep Kit for Illumina (New England Biolabs, Ipswich, MA, USA) and sequenced on a flow cell of the Illumina HiSeq 2500 platform (Illumina, Inc., San Diego, CA, USA). The transcripts raw data have been deposited in the NCBI database with a project number PRJNA784034 (https://dataview.ncbi.nlm.nih.gov/object/PRJNA784034?reviewer=v79bcipc97tajk7ppuam33d88t, accessed on 21 February 2022).

### 4.6. RNA Isolation, cDNA Synthesis, and qRT-PCR

Total RNA was extracted from *Z. bungeanum* leaf samples using the Tiangen RNA Pure kit for plants (Tiangen, Beijing, China). The integrity, purity, and concentration of the total RNA were determined using a NanoDrop 2000 spectrophotometer (Thermo Scientific, Wilmington, DE, USA). DNA-free RNA was used for first-strand cDNA synthesis with the PrimeScript RT reagent kit with gDNA Eraser (Takara Biotechnology Inc., Dalian, China). qRT-PCR was performed on a CFX96 Real-Time System (Bio-Rad Laboratories, Inc., Hercules, CA, USA) with using SYBR^®^ Green Premix *Pro Taq* HS qPCR Kit (Accurate Biotechnology Co., Ltd., Hunan, China). The reaction protocol was as follows: 1 cycle at 98 °C for 30 s; 38 cycles at 95 °C for 5 s, 56 °C for 30 s, and 72 °C for 30 s. Primer Premier 6.0 (PREMIER Biosoft, CA, USA) was used to design the specific primers for qRT-PCR (Table S5). qRT-PCR was performed with three biological replicates, and relative gene expression was calculated by the 2^−ΔΔCt^ method [70]. The two reference genes *ZbUBA* and *ZbUBQ* were used to standardize the expression levels [71].

### 4.7. Co-expression Network and Protein–Protein Interaction Network Analysis of ZbNAC Genes

Co-expression relationships were analyzed based on FPKM values from RNA-seq using Cytohubba in Cytoscape (version 3.9.1) with a threshold of 0.25. The protein–protein interaction network was predicted with STRING 11.5 (https://cn.string-db.org/, accessed on 22 February, 2022) using the NAC proteins from *Arabidopsis* that were homologous to the NAC proteins in *Z. bungeanum*.

## 5. Conclusions

In this research, 109 ZbNAC TFs were identified in *Z. bungeanum* and were classified into four subgroups with NAC proteins from *Arabidopsis*. These NAC TF genes were unevenly distributed on 46 chromosomes. Conserved motifs and gene structures showed similarity in NAC TFs clustering together in phylogeny tree. The inter-species synteny analysis of NAC genes revealed the close phylogeny relationship between *Z. bungeanum* and *C. sinensis*. Meanwhile, promoter *cis*-element analysis implied the multiple functions of NAC in abiotic stresses. Moreover, the expression levels were significantly stimulated in most of *ZbNAC*s in both *Z. bungeanum* cultivars under progressive drought stress, therein five *ZbNAC* genes (*ZbNAC007*, *ZbNAC018*, *ZbNAC047*, *ZbNAC072*, and *ZbNAC079*) were potential candidate genes for drought tolerance in *Z. bungeanum*. In general, the genome-wide analysis of NAC family was first performed in *Z. bungeanum* and the expression pattern of *ZbNAC* genes was carefully explored under drought in this study. The results improved our understanding of NAC family in plants. Additionally, these candidate *ZbNAC* genes provide pivotal clues for molecular breeding of *Z. bungeanum* in the future.

## Figures and Tables

**Table 1:** Duplications of NAC genes in *Z. bungeanum*.

Gene 1	Gene 2	Ka	Ks	*Ka*/*Ks*	Duplication Type
*ZbNAC001*	*ZbNAC002*	0.10	0.43	0.24	tandem
*ZbNAC001*	*ZbNAC078*	0.15	0.40	0.37	segmental
*ZbNAC003*	*ZbNAC069*	0.09	0.27	0.31	segmental
*ZbNAC003*	*ZbNAC058*	0.11	0.30	0.35	segmental
*ZbNAC004*	*ZbNAC057*	0.07	0.36	0.20	segmental
*ZbNAC006*	*ZbNAC021*	0.06	0.30	0.21	segmental
*ZbNAC009*	*ZbNAC010*	0.01	0.03	0.18	segmental
*ZbNAC009*	*ZbNAC013*	0.09	0.29	0.32	segmental
*ZbNAC010*	*ZbNAC012*	0.09	0.26	0.35	segmental
*ZbNAC024*	*ZbNAC035*	0.46	1.06	0.43	segmental
*ZbNAC024*	*ZbNAC028*	0.47	0.73	0.64	segmental
*ZbNAC025*	*ZbNAC026*	0.20	0.27	0.74	segmental
*ZbNAC026*	*ZbNAC027*	0.21	0.26	0.82	tandem
*ZbNAC026*	*ZbNAC036*	0.47	0.65	0.72	segmental
*ZbNAC027*	*ZbNAC028*	0.20	0.17	1.22	tandem
*ZbNAC028*	*ZbNAC029*	0.52	0.84	0.61	tandem
*ZbNAC029*	*ZbNAC030*	0.36	0.53	0.68	tandem
*ZbNAC035*	*ZbNAC036*	0.02	0.04	0.64	segmental
*ZbNAC038*	*ZbNAC031*	0.11	0.35	0.30	segmental
*ZbNAC039*	*ZbNAC033*	0.07	0.22	0.34	segmental
*ZbNAC041*	*ZbNAC097*	0.11	0.33	0.32	segmental
*ZbNAC043*	*ZbNAC045*	0.07	0.22	0.31	segmental
*ZbNAC044*	*ZbNAC101*	0.06	0.18	0.33	segmental
*ZbNAC046*	*ZbNAC092*	0.08	0.36	0.23	segmental
*ZbNAC049*	*ZbNAC063*	0.09	0.19	0.47	segmental
*ZbNAC049*	*ZbNAC050*	0.01	0.01	1.13	segmental
*ZbNAC050*	*ZbNAC063*	0.10	0.21	0.49	segmental
*ZbNAC053*	*ZbNAC090*	0.04	0.19	0.20	segmental
*ZbNAC058*	*ZbNAC069*	0.10	0.27	0.38	segmental
*ZbNAC059*	*ZbNAC090*	0.04	0.17	0.24	segmental
*ZbNAC064*	*ZbNAC088*	0.17	0.27	0.62	segmental
*ZbNAC064*	*ZbNAC087*	0.08	0.12	0.71	segmental
*ZbNAC066*	*ZbNAC089*	0.09	0.16	0.54	segmental
*ZbNAC068*	*ZbNAC100*	0.07	0.25	0.26	segmental
*ZbNAC068*	*ZbNAC069*	0.00	0.01	0.35	segmental
*ZbNAC071*	*ZbNAC012*	0.07	0.28	0.26	segmental
*ZbNAC071*	*ZbNAC009*	0.08	0.29	0.26	segmental
*ZbNAC071*	*ZbNAC013*	0.07	0.28	0.26	segmental
*ZbNAC072*	*ZbNAC074*	0.01	0.03	0.27	segmental
*ZbNAC072*	*ZbNAC007*	0.09	0.29	0.31	segmental
*ZbNAC073*	*ZbNAC011*	0.14	0.32	0.45	segmental
*ZbNAC074*	*ZbNAC007*	0.09	0.27	0.33	segmental
*ZbNAC076*	*ZbNAC099*	0.00	0.04	0.11	segmental
*ZbNAC105*	*ZbNAC028*	0.52	1.18	0.44	segmental
*ZbNAC105*	*ZbNAC024*	0.24	0.39	0.60	segmental
*ZbNAC106*	*ZbNAC037*	0.06	0.34	0.16	segmental
*ZbNAC107*	*ZbNAC108*	0.04	0.05	0.74	segmental

## Data Availability

All data in the present study are available in the public database as described in the Materials and Methods section.

## Supplementary Materials

The following supporting information can be downloaded at: https://www.mdpi.com/article/10.3390/ijms23094769/s1.

## References

[1] Riechmann J.L., Heard J., Martin G., Reuber L., Jiang C., Keddie J., Adam L., Pineda O., Ratcliffe O.J., Samaha R.R. (2000). Arabidopsis Transcription Factors: Genome-Wide Comparative Analysis Among Eukaryotes. Science.

[2] Puranik S., Sahu P.P., Srivastava P.S., Prasad M. (2012). NAC proteins: Regulation and role in stress tolerance. Trends Plant Sci..

[3] Olsen A.N., Ernst H.A., Leggio L.L., Skriver K. (2005). NAC transcription factors: Structurally distinct, functionally diverse. Trends Plant Sci..

[4] Zhang L., Yao L., Zhang N., Yang J., Zhu X., Tang X., Calderón-Urrea A., Si H. (2018). Lateral Root Development in Potato Is Mediated by Stu-mi164 Regulation of NAC Transcription Factor. Front. Plant Sci..

[5] Kucukoglu M. (2020). A novel NAC domain transcription factor XVP controls the balance of xylem formation and cambial cell divisions. New Phytol..

[6] El Mannai Y., Akabane K., Hiratsu K., Satoh-Nagasawa N., Wabiko H. (2017). The NAC Transcription Factor Gene OsY37 (ONAC011) Promotes Leaf Senescence and Accelerates Heading Time in Rice. Int. J. Mol. Sci..

[7] Liu G.-S., Li H.-L., Grierson D., Fu D.-Q. (2022). NAC Transcription Factor Family Regulation of Fruit Ripening and Quality: A Review. Cells.

[8] Wang J., Wang Y., Zhang J., Ren Y., Li M., Tian S., Yu Y., Zuo Y., Gong G., Zhang H. (2021). The NAC transcription factor ClNAC68 positively regulates sugar content and seed development in watermelon by repressing ClINV and ClGH3.6. Hortic. Res..

[9] Li M., Chen R., Jiang Q., Sun X., Zhang H., Hu Z. (2021). GmNAC06, a NAC domain transcription factor enhances salt stress tolerance in soybean. Plant Mol. Biol..

[10] Wang Z., Ni L., Liu D., Fu Z., Hua J., Lu Z., Liu L., Yin Y., Li H., Gu C. (2022). Genome-Wide Identification and Characterization of NAC Family in *Hibiscus hamabo* Sieb. et Zucc. under Various Abiotic Stresses. Int. J. Mol. Sci..

[11] Guo W., Zhang J., Zhang N., Xin M., Peng H., Hu Z., Ni Z., Du J. (2015). The Wheat NAC Transcription Factor TaNAC2L Is Regulated at the Transcriptional and Post-Translational Levels and Promotes Heat Stress Tolerance in Transgenic Arabidopsis. PLoS ONE.

[12] Yan H., Ma G., da Silva J.A.T., Qiu L., Xu J., Zhou H., Wei M., Xiong J., Li M., Zhou S. (2021). Genome-Wide Identification and Analysis of NAC Transcription Factor Family in Two Diploid Wild Relatives of Cultivated Sweet Potato Uncovers Potential NAC Genes Related to Drought Tolerance. Front. Genet..

[13] Corso M., Vannozzi A., Maza E., Vitulo N., Meggio F., Pitacco A., Telatin A., D’Angelo M., Feltrin E., Negri A.S. (2015). Comprehensive transcript profiling of two grapevine rootstock genotypes contrasting in drought susceptibility links the phenylpropanoid pathway to enhanced tolerance. J. Exp. Bot..

[14] Jiang D., Zhou L., Chen W., Ye N., Xia J., Zhuang C. (2019). Overexpression of a microRNA-targeted NAC transcription factor improves drought and salt tolerance in Rice via ABA-mediated pathways. Rice.

[15] Mao H., Li S., Chen B., Jian C., Mei F., Zhang Y., Li F., Chen N., Li T., Du L. (2021). Variation in cis-regulation of a NAC transcription factor contributes to drought tolerance in wheat. Mol. Plant.

[16] Yu M., Liu J., Du B., Zhang M., Wang A., Zhang L. (2021). NAC Transcription Factor PwNAC11 Activates *ERD1* by Interaction with ABF3 and DREB2A to Enhance Drought Tolerance in Transgenic *Arabidopsis*. Int. J. Mol. Sci..

[17] Srivastava R., Kobayashi Y., Koyama H., Sahoo L. (2022). Overexpression of cowpea NAC transcription factors promoted growth and stress tolerance by boosting photosynthetic activity in Arabidopsis. Plant Sci..

[18] Feng S., Liu Z., Hu Y., Tian J., Yang T., Wei A. (2020). Genomic analysis reveals the genetic diversity, population structure, evolutionary history and relationships of Chinese pepper. Hortic. Res..

[19] Ma Y., Li X., Hou L.-X., Wei A.-Z. (2019). Extraction solvent affects the antioxidant, antimicrobial, cholinesterase and HepG2 human hepatocellular carcinoma cell inhibitory activities of Zanthoxylum bungeanum pericarps and the major chemical components. Ind. Crop. Prod..

[20] Li M., Hou L., Liu S., Zhang C., Yang W., Pang X., Li Y. (2021). Genome-wide identification and expression analysis of NAC transcription factors in Ziziphus jujuba Mill. reveal their putative regulatory effects on tissue senescence and abiotic stress responses. Ind. Crop. Prod..

[21] Luoni S.A.B., Cenci A., Moschen S., Nicosia S., Radonic L.M., García J.V.S.Y., Langlade N.B., Vile D., Rovere C.V., Fernandez P. (2021). Genome-wide and comparative phylogenetic analysis of senescence-associated NAC transcription factors in sunflower (Helianthus annuus). BMC Genom..

[22] Li X., Cai K., Pei X., Li Y., Hu Y., Meng F., Song X., Tigabu M., Ding C., Zhao X. (2021). Genome-Wide Identification of NAC Transcription Factor Family in *Juglans mandshurica* and Their Expression Analysis during the Fruit Development and Ripening. Int. J. Mol. Sci..

[23] Li P., Peng Z., Xu P., Tang G., Ma C., Zhu J., Shan L., Wan S. (2021). Genome-Wide Identification of NAC Transcription Factors and Their Functional Prediction of Abiotic Stress Response in Peanut. Front. Genet..

[24] Feng S., Liu Z., Cheng J., Li Z., Tian L., Liu M., Yang T., Liu Y., Liu Y., Dai H. (2021). Zanthoxylum-specific whole genome duplication and recent activity of transposable elements in the highly repetitive paleotetraploid Z. bungeanum genome. Hortic. Res..

[25] Eshao H.-B., Ewang H., Etang X. (2015). NAC transcription factors in plant multiple abiotic stress responses: Progress and prospects. Front. Plant Sci..

[26] Yang Z., Nie G., Feng G., Han J., Huang L., Zhang X. (2021). Genome-wide identification, characterization, and expression analysis of the NAC transcription factor family in orchardgrass (*Dactylis glomerata* L.). BMC Genom..

[27] Wei S., Gao L., Zhang Y., Zhang F., Yang X., Huang D. (2016). Genome-wide investigation of the NAC transcription factor family in melon (*Cucumis melo* L.) and their expression analysis under salt stress. Plant Cell Rep..

[28] Liu M., Ma Z., Sun W., Huang L., Wu Q., Tang Z., Bu T., Li C., Chen H. (2019). Genome-wide analysis of the NAC transcription factor family in Tartary buckwheat (*Fagopyrum tataricum*). BMC Genom..

[29] Hu W., Wei Y., Xia Z., Yan Y., Hou X., Zou M., Lu C., Wang W., Peng M. (2015). Genome-Wide Identification and Expression Analysis of the NAC Transcription Factor Family in Cassava. PLoS ONE.

[30] Nuruzzaman M., Manimekalai R., Sharoni A.M., Satoh K., Kondoh H., Ooka H., Kikuchi S. (2010). Genome-wide analysis of NAC transcription factor family in rice. Gene.

[31] Shiriga K., Sharma R., Kumar K., Yadav S.K., Hossain F., Thirunavukkarasu N. (2014). Genome-wide identification and expression pattern of drought-responsive members of the NAC family in maize. Meta Gene.

[32] Le D.T., Nishiyama R., Watanabe Y., Mochida K., Yamaguchi-Shinozaki K., Shinozaki K., Tran L.-S.P. (2011). Genome-Wide Survey and Expression Analysis of the Plant-Specific NAC Transcription Factor Family in Soybean During Development and Dehydration Stress. DNA Res..

[33] Bowers J., Chapman B., Rong J., Paterson A.H. (2003). Unravelling angiosperm genome evolution by phylogenetic analysis of chromosomal duplication events. Nature.

[34] Mohanta T.K., Yadav D., Khan A., Hashem A., Tabassum B., Khan A.L., Abd_Allah E.F., Al-Harrasi A. (2020). Genomics, molecular and evolutionary perspective of NAC transcription factors. PLoS ONE.

[35] Cannon S.B., Mitra A., Baumgarten A., Young N.D., May G. (2004). The roles of segmental and tandem gene duplication in the evolution of large gene families in Arabidopsis thaliana. BMC Plant Biol..

[36] Shan Z., Jiang Y., Li H., Guo J., Dong M., Zhang J., Liu G. (2020). Genome-wide analysis of the NAC transcription factor family in broomcorn millet (*Panicum miliaceum* L.) and expression analysis under drought stress. BMC Genom..

[37] Li B., Fan R., Yang Q., Hu C., Sheng O., Deng G., Dong T., Li C., Peng X., Bi F. (2020). Genome-Wide Identification and Characterization of the NAC Transcription Factor Family in Musa Acuminata and Expression Analysis during Fruit Ripening. Int. J. Mol. Sci..

[38] Lynch M., Conery J.S. (2000). The Evolutionary Fate and Consequences of Duplicate Genes. Science.

[39] Kandul N.P., Noor M.A. (2009). Large introns in relation to alternative splicing and gene evolution: A case study of Drosophila bruno-3. BMC Genet..

[40] Singh S., Kudapa H., Garg V., Varshney R.K. (2021). Comprehensive analysis and identification of drought-responsive candidate NAC genes in three semi-arid tropics (SAT) legume crops. BMC Genom..

[41] Li W., Zeng Y., Yin F., Wei R., Mao X. (2021). Genome-wide identification and comprehensive analysis of the NAC transcription factor family in sunflower during salt and drought stress. Sci. Rep..

[42] Zhang S., Xu R., Gao Z., Chen C., Jiang Z., Shu H. (2013). A genome-wide analysis of the expansin genes in Malus × Domestica. Mol. Genet. Genom..

[43] Nie G., Yang Z., He J., Liu A., Chen J., Wang S., Wang X., Feng G., Li D., Peng Y. (2021). Genome-Wide Investigation of the NAC Transcription Factor Family in Miscanthus sinensis and Expression Analysis Under Various Abiotic Stresses. Front. Plant Sci..

[44] Wu R., Duan L., Pruneda-Paz J.L., Oh D.-H., Pound M., Kay S., Dinneny J.R. (2018). The 6xABRE Synthetic Promoter Enables the Spatiotemporal Analysis of ABA-Mediated Transcriptional Regulation. Plant Physiol..

[45] Ehong Y., Ezhang H., Ehuang L., Eli D., Esong F. (2016). Overexpression of a Stress-Responsive NAC Transcription Factor Gene ONAC022 Improves Drought and Salt Tolerance in Rice. Front. Plant Sci..

[46] Han D., Du M., Zhou Z., Wang S., Li T., Han J., Xu T., Yang G. (2020). Han Overexpression of a Malus baccata NAC Transcription Factor Gene MbNAC25 Increases Cold and Salinity Tolerance in Arabidopsis. Int. J. Mol. Sci..

[47] Fu J., Wu H., Ma S., Xiang D., Liu R., Xiong L. (2017). OsJAZ1 Attenuates Drought Resistance by Regulating JA and ABA Signaling in Rice. Front. Plant Sci..

[48] Liu W., Jiang Y., Jin Y., Wang C., Yang J., Qi H. (2021). Drought-induced ABA, H_2_O_2_ and JA positively regulate CmCAD genes and lignin synthesis in melon stems. BMC Plant Biol..

[49] Aroca R., Alguacil M.D.M., Vernieri P., Ruiz-Lozano J.M. (2008). Plant Responses to Drought Stress and Exogenous ABA Application are Modulated Differently by Mycorrhization in Tomato and an ABA-deficient Mutant (Sitiens). Microb. Ecol..

[50] Jiang Y., Ye J., Li S. (2017). Niinemets, Ülo Methyl jasmonate-induced emission of biogenic volatiles is biphasic in cucumber: A high-resolution analysis of dose dependence. J. Exp. Bot..

[51] Liu T., Li C.-X., Zhong J., Shu D., Luo D., Li Z.-M., Zhou J.-Y., Yang J., Tan H., Ma X.-R. (2021). Exogenous 1’,4’-*trans*-Diol-ABA Induces Stress Tolerance by Affecting the Level of Gene Expression in Tobacco (*Nicotiana tabacum* L.). Int. J. Mol. Sci..

[52] Yuan C., Li C., Lu X., Zhao X., Yan C., Wang J., Sun Q., Shan S. (2020). Comprehensive genomic characterization of NAC transcription factor family and their response to salt and drought stress in peanut. BMC Plant Biol..

[53] Diao W., Snyder J.C., Wang S., Liu J., Pan B., Guo G., Ge W., Dawood M.H.S.A. (2018). Genome-Wide Analyses of the NAC Transcription Factor Gene Family in Pepper (*Capsicum annuum* L.): Chromosome Location, Phylogeny, Structure, Expression Patterns, Cis-Elements in the Promoter, and Interaction Network. Int. J. Mol. Sci..

[54] Malik W.A., Afzal M., Chen X., Cui R., Lu X., Wang S., Wang J., Mahmood I., Ye W. (2021). Systematic analysis and comparison of ABC proteins superfamily confer structural, functional and evolutionary insights into four cotton species. Ind. Crop. Prod..

[55] Hou Q., Qiu Z., Wen Z., Zhang H., Li Z., Hong Y., Qiao G., Wen X. (2021). Genome-Wide Identification of ARF Gene Family Suggests a Functional Expression Pattern during Fruitlet Abscission in *Prunus avium* L. Int. J. Mol. Sci..

[56] Wang M., Chen B., Zhou W., Xie L., Wang L., Zhang Y., Zhang Q. (2021). Genome-wide identification and expression analysis of the AT-hook Motif Nuclear Localized gene family in soybean. BMC Genom..

[57] Zhang H., Zhu J., Gong Z., Zhu J.-K. (2021). Abiotic stress responses in plants. Nat. Rev. Genet..

[58] Danquah A., de Zelicourt A., Colcombet J., Hirt H. (2014). The role of ABA and MAPK signaling pathways in plant abiotic stress responses. Biotechnol. Adv..

[59] Wong M.M., Bhaskara G.B., Wen T.-N., Lin W.-D., Nguyen T.T., Chong G.L., Verslues P.E. (2019). Phosphoproteomics of *Arabidopsis* Highly ABA-Induced1 identifies AT-Hook– Like10 phosphorylation required for stress growth regulation. Proc. Natl. Acad. Sci. USA.

[60] de Zelicourt A., Colcombet J., Hirt H. (2016). The Role of MAPK Modules and ABA during Abiotic Stress Signaling. Trends Plant Sci..

[61] Sakuraba Y., Kim Y.-S., Han S.-H., Lee B.-D., Paek N.-C. (2015). The Arabidopsis Transcription Factor NAC016 Promotes Drought Stress Responses by Repressing *AREB1* Transcription through a Trifurcate Feed-Forward Regulatory Loop Involving NAP. Plant Cell.

[62] Jiang H., Tang B., Xie Z., Nolan T., Ye H., Song G., Walley J., Yin Y. (2019). GSK 3-like kinase BIN 2 phosphorylates RD 26 to potentiate drought signaling in Arabidopsis. Plant J..

[63] Watanabe N., Lam E. (2004). Recent advance in the study of caspase-like proteases and Bax inhibitor-1 in plants: Their possible roles as regulator of programmed cell death. Mol. Plant Pathol..

[64] Petrov V., Hille J., Mueller-Roeber B., Gechev T.S. (2015). ROS-mediated abiotic stress-induced programmed cell death in plants. Front. Plant Sci..

[65] Prakash A., Jeffryes M., Bateman A., Finn R.D. (2017). The HMMER Web Server for Protein Sequence Similarity Search. Curr. Protoc. Bioinform..

[66] Chou K.-C., Shen H.-B. (2008). Cell-PLoc: A package of Web servers for predicting subcellular localization of proteins in various organisms. Nat. Protoc..

[67] Chen C., Chen H., Zhang Y., Thomas H.R., Frank M.H., He Y., Xia R. (2020). TBtools: An Integrative Toolkit Developed for Interactive Analyses of Big Biological Data. Mol. Plant.

[68] Wang Y., Tang H., DeBarry J.D., Tan X., Li J., Wang X., Lee T.-H., Jin H., Marler B., Guo H. (2012). *MCScanX*: A toolkit for detection and evolutionary analysis of gene synteny and collinearity. Nucleic Acids Res..

[69] Ouyang S., Zhu W., Hamilton J., Lin H., Campbell M., Childs K., Thibaud-Nissen F., Malek R.L., Lee Y., Zheng L. (2006). The TIGR Rice Genome Annotation Resource: Improvements and new features. Nucleic Acids Res..

[70] Schmittgen T.D., Livak K.J. (2008). Analyzing real-time PCR data by the comparative *C*_T_ method. Nat. Protoc..

[71] Fei X., Shi Q., Yang T., Fei Z., Wei A. (2018). Expression Stabilities of Ten Candidate Reference Genes for RT-qPCR in Zanthoxylum bungeanum Maxim. Molecules.

