# Supplementary material for: Genome-Wide Identification of the NAC Gene Family in Zanthoxylum bungeanum and Their Transcriptional Responses to Drought Stress
Source: Int J Mol Sci. 2022 Apr 26;23(9):4769. doi: 10.3390/ijms23094769 (PMC9103986; doi:10.3390/ijms23094769)
Supplement: Supplementary file 1 [file ijms-23-04769-s001.zip › Supplementary materials/Supplementary figures.pdf]

Supplementary Figures

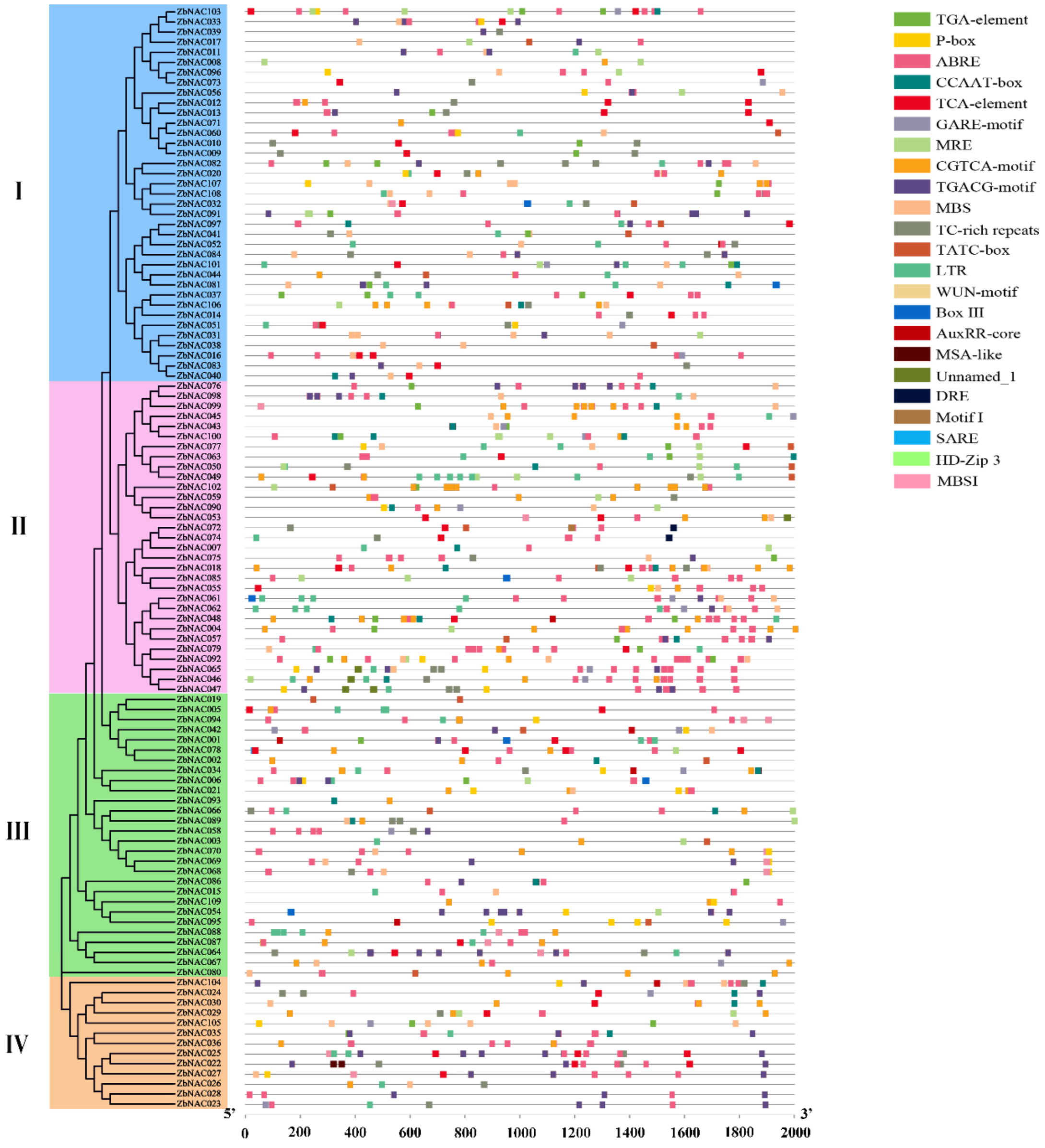

Figure S1. *cis*-element positions in *ZbNAC* promoters.

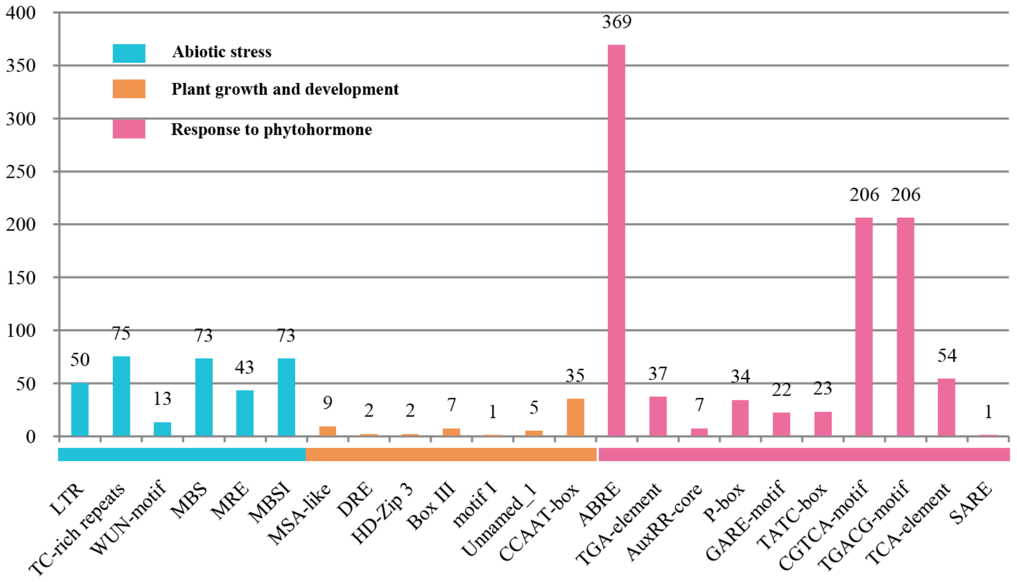

Figure S2. The total amount of each *cis*-element in the promoter regions of 108 *ZbNAC* genes.
